# Correction: A smile is all you need: predicting limiting activity coefficients from SMILES with natural language processing

**DOI:** 10.1039/d4dd90045f

**Published:** 2024-10-14

**Authors:** Benedikt Winter, Clemens Winter, Johannes Schilling, André Bardow

**Affiliations:** a Energy and Process System Engineering, ETH Zürich Tannenstrasse 3 8092 Zürich Switzerland abardow@ethz.ch; b OpenAI 3180 18TH St. San Francisco CA 94110 USA

## Abstract

Correction for ‘A smile is all you need: predicting limiting activity coefficients from SMILES with natural language processing’ by Benedikt Winter *et al.*, *Digital Discovery*, 2022, **1**, 859–869, https://doi.org/10.1039/D2DD00058J.

It has come to our attention that certain resources mentioned in the data availability statement of our previously published article need to be taken offline due to legal issues. We would like to provide the following corrections to the data availability statement:

## Corrected data availability statement

Due to legal issues, the following resources are no longer available: Code, datasets, and trained models. No additional data is available beyond the manuscript and its ESI. For any questions or further information, please contact the corresponding author.

The authors apologise for these errors and any consequent inconvenience to authors and readers.

The Royal Society of Chemistry apologises for these errors and any consequent inconvenience to authors and readers.

